# Contribution of Interleukin-22 Binding Protein to the Development of Allergen-Induced Airway Hyperresponsiveness

**DOI:** 10.3390/ijms27135909

**Published:** 2026-06-30

**Authors:** Ryota Sunami, Hisao Higo, Satoru Senoo, Akihiko Taniguchi, Taichi Ozeki, Naoki Nakamura, Ayako Morita, Shusei Yamamoto, Tomoki Kitazoe, Yumi Inukai, Takashi Kanaya, Hiroshi Ohno, Katsuyuki Kiura, Yoshinobu Maeda, Nobuaki Miyahara

**Affiliations:** 1 Department of Hematology, Oncology, and Respiratory Medicine, Graduate School of Medicine, Dentistry, and Pharmaceutical Sciences, Okayama University, 2-5-1 Shikata-cho, Kita-ku, Okayama 700-8558, Japanshuttle1126@gmail.com (S.S.); me20020@s.okayama-u.ac.jp (T.O.); n.naoki.0126@gmail.com (N.N.); pyo1817m@s.okayama-u.ac.jp (A.M.); pirr3fol@s.okayama-u.ac.jp (Y.I.); yosmaeda@md.okayama-u.ac.jp (Y.M.); 2 Department of Respiratory Medicine, Okayama University Hospital, 2-5-1 Shikata-cho, Kita-ku, Okayama 700-8558, Japan; prea4jsb@s.okayama-u.ac.jp (H.H.);; 3 Department of Medical Laboratory Science, Faculty of Health Sciences, Okayama University, 2-5-1 Shika-ta-cho, Kita-ku, Okayama 700-8558, Japan; s-yamamoto@okayama-u.ac.jp (S.Y.);; 4 Department of Medical Laboratory Science, Faculty of Health Sciences, Junshin Gakuen University, Fukuoka 815-8510, Japan; 5 Laboratory for Intestinal Ecosystem, RIKEN Center for Integrative Medical Sciences, Yokohama 230-004, Japanhiroshi.ohno@riken.jp (H.O.)

**Keywords:** airway hyperresponsiveness, IL-22, airway inflammation

## Abstract

Interleukin-22 binding protein (IL-22BP) is a soluble decoy receptor that competitively inhibits IL-22 by preventing its interaction with the IL-22 receptor. Although the IL-22 receptor is primarily expressed on non-hematopoietic cells, such as airway epithelial cells, the role of IL-22BP in the pathogenesis of asthma remains uncertain. We observed that IL-22BP was upregulated in the airways of wild-type (WT) mice intranasally sensitized and challenged with house dust mite (HDM) extract. To directly elucidate the function of IL-22BP in allergic airway responses, IL-22BP-deficient (IL-22BP−/−) and WT mice were sensitized and challenged with HDM, and airway responses were systematically assessed. IL-22BP−/− mice exhibited significantly lower airway hyperresponsiveness (AHR) compared to WT mice following sensitization and challenge with HDM. In contrast, eosinophil counts in bronchoalveolar lavage (BAL) fluid did not differ significantly between the two groups. Similarly, levels of interleukin (IL)-4, IL-5, IL-6, IL-13, IL-17A, and keratinocyte chemoattractant (KC) in BAL fluid were comparable between WT and IL-22BP−/− mice. Notably, IL-22 levels in lung homogenates were significantly higher in IL-22BP−/− mice than in WT mice after sensitization and challenge with HDM. These findings suggest that inhibition of IL-22BP attenuates the development of allergen-induced AHR, an effect likely mediated through enhanced IL-22 activity rather than alterations in airway inflammation or type 2 cytokine production.

## 1. Introduction

Bronchial asthma is characterized by airway hyperresponsiveness (AHR) and airway inflammation. AHR is significantly associated with inflammation, which results from the accumulation of activated eosinophils and T cells at the site of injury [1,2]. In addition to T helper type 2 (Th2) cells, innate lymphoid cells, which secrete interleukin (IL)-5 and IL-13, play critical roles in the development of allergic airway inflammation and AHR [3,4].

IL-22, a member of the IL-10 cytokine family, plays a critical role in resolving lung infection and injury [5]. It is produced by CD4^+^ T cells, innate lymphoid cells, and gamma-delta T cells. IL-22 activates signal transducer and activator of transcription 3 in non-immune cells, such as airway epithelial cells, promoting tissue homeostasis and immune surveillance [6]. Recent studies suggest that IL-22 can attenuate allergic airway inflammation by suppressing Th2- and Th17-mediated immune responses [7]. In contrast, previous studies have reported opposing effects of IL-22 on allergic airway responses [8,9]. Therefore, the role of IL-22 in allergic airway inflammation remains uncertain.

The IL-22 receptor consists of the heterodimers IL-22Rα1 and IL-10R2, which are expressed on non-hematopoietic cells, such as airway epithelial cells [7]. IL-22 binding protein (IL-22BP; also known as IL-22Rα2) functions as a soluble decoy receptor that binds IL-22 and competitively inhibits its activity [7]. IL-22BP is primarily secreted by dendritic cells [10]. Recent studies have emphasized important roles of IL-22BP in infection and respiratory diseases, including acute lung injury [11,12]. However, its contribution to the pathogenesis of allergic airway responses remains uncertain.

We investigated the role of IL-22BP in a murine model of allergen-induced airway responses. Allergen sensitization and challenge resulted in increased IL-22BP expression. Compared with wild-type (WT) mice, IL-22BP-deficient (IL-22BP−/−) mice exhibited significantly reduced AHR, indicating a key role for IL-22BP in the development of AHR. These findings suggest that targeting IL-22BP may represent a potential strategy for controlling allergen-induced AHR.

## 2. Results

### 2.1. IL-22BP Levels in BAL Fluid and Serum

To investigate the role of IL-22BP in allergen-induced airway responses, WT mice were sensitized and challenged with HDM, and IL-22BP levels were measured 24 h after the final challenge.

As expected, sensitized and challenged WT mice exhibited significantly higher AHR compared with non-sensitized, non-challenged controls (Supplementary Figure S1). Moreover, they demonstrated increased eosinophil counts in BAL fluid (Supplementary Figure S2). These findings confirm the successful establishment of the HDM-induced allergic airway disease model used in this study.

**Figure 1:**
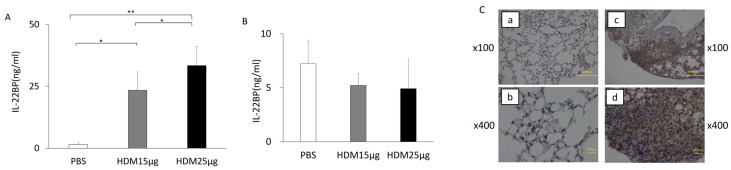
IL-22BP levels in BAL fluid and serum following sensitization and challenge. (**A**,**B**) IL-22BP levels in BAL fluid and serum were measured by ELISA, as described in Materials and Methods. (**C**) Immunohistochemical staining of IL-22BP in lung tissue from PBS-sensitized and -challenged WT mice (**a**,**b**) and HDM-sensitized and -challenged (25 µg) WT mice (**c**,**d**), shown at different magnifications ((**a**,**c**) ×100; (**b**,**d**) ×400). IL-22BP expression was evaluated 24 h after the last challenge. IL-22BP-positive cells are indicated by brown staining. Data are presented as means ± SE (*n* = 4–10 per group). * *p* < 0.05, ** *p* < 0.01 in all figures. IL-22BP, interleukin-22 binding protein; HDM, house dust mite; ELISA, enzyme-linked immunosorbent assay; WT, wild-type; PBS, phosphate-buffered saline; BAL, bronchoalveolar lavage; SE, standard error.

IL-22BP levels in BAL fluid were significantly elevated in sensitized and challenged WT mice compared to controls and increased in a dose-dependent manner with HDM challenge (Figure 1A). In contrast, IL-22BP levels in serum exhibited a decreasing trend in sensitized and challenged WT mice compared to controls, but the differences were not statistically significant (Figure 1B).

### 2.2. IL-22BP Expression in Lung Tissue

IL-22BP expression in WT lung tissue was evaluated by immunohistochemistry 24 h after the final HDM challenge. Relatively few IL-22BP-positive cells were detected in non-sensitized, non-challenged mice (Figure 1(Ca,Cb)). In contrast, IL-22BP expression was observed predominantly in alveolar walls, vascular endothelial cells, and inflammatory cells surrounding the bronchi in sensitized and challenged mice (Figure 1(Cc,Cd)).

### 2.3. IL-22BP−/− Mice Develop Reduced AHR Following Sensitization and Challenge

**Figure 2:**
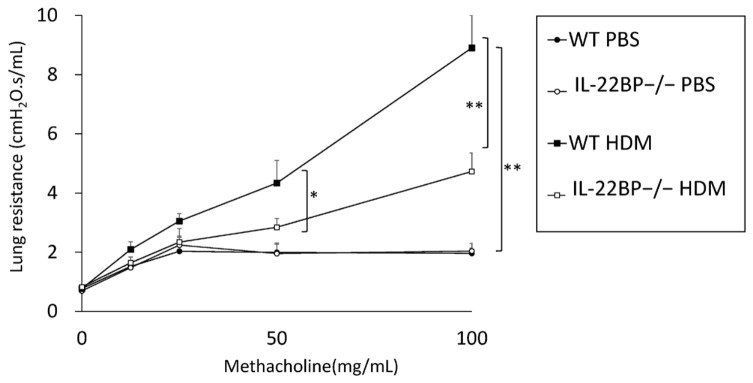
Reduced AHR in IL-22BP−/− mice following sensitization and challenge. WT and IL-22−/− mice were sensitized and challenged with HDM. In total, 24 h after the last challenge, lung resistance was measured in response to increasing concentrations of inhaled methacholine, as described in Materials and Methods. Data are presented as means ± SE (*n* = 4–10 per group). * *p* < 0.05, ** *p* < 0.01. IL-22BP, interleukin-22 binding protein; HDM, house dust mite; AHR, airway hyperresponsiveness; WT, wild-type; SE, standard error.

AHR was assessed 24 h after the final HDM challenge in WT and IL-22BP−/− mice. Sensitized and challenged WT mice exhibited a significant increase in lung resistance compared with non-sensitized, non-challenged controls (Figure 2). In contrast, sensitized and challenged IL-22BP−/− mice exhibited a significantly attenuated increase in lung resistance relative to sensitized and challenged WT mice; however, their responses remained significantly higher than those of non-sensitized, non-challenged IL-22BP−/− mice.

### 2.4. Airway Inflammation in IL-22BP−/− Mice

**Figure 3:**
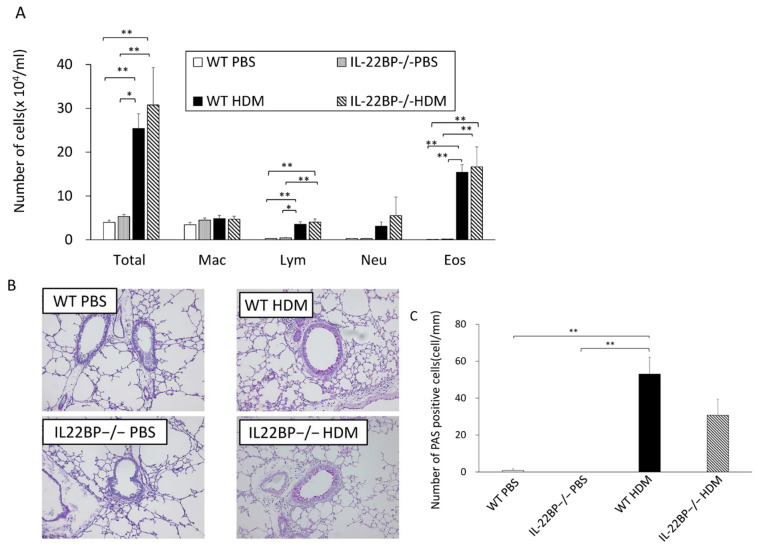
Airway inflammation in WT and IL-22BP−/− mice following sensitization and challenge. (**A**) Cellular composition in BAL fluid of WT and IL-22BP−/− mice. (**B**) Goblet cell metaplasia in the airways of WT and IL-22BP−/− mice. (**C**) Quantification of goblet cell metaplasia in PAS-stained sections, as described in Materials and Methods. Data are presented as means ± SE (*n* = 4–10 per group). * *p* < 0.05, ** *p* < 0.01. IL-22BP, interleukin-22 binding protein; PAS, periodic acid–Schiff; WT, wild-type; SE, standard error; BAL, bronchoalveolar lavage.

BAL fluid analysis revealed a significant increase in eosinophils in sensitized and challenged mice compared to non-sensitized, non-challenged controls. Sensitized and challenged IL-22BP−/− mice demonstrated increased eosinophil counts, with no significant differences compared to their WT counterparts (Figure 3A). Similarly, the numbers of PAS-positive goblet cells did not differ significantly between IL-22BP−/− mice and WT mice following HDM sensitization and challenge (Figure 3B,C).

### 2.5. Airway Cytokine Levels

**Figure 4:**
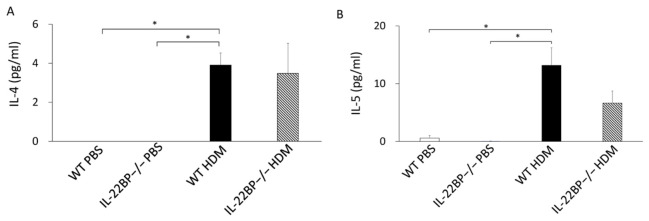

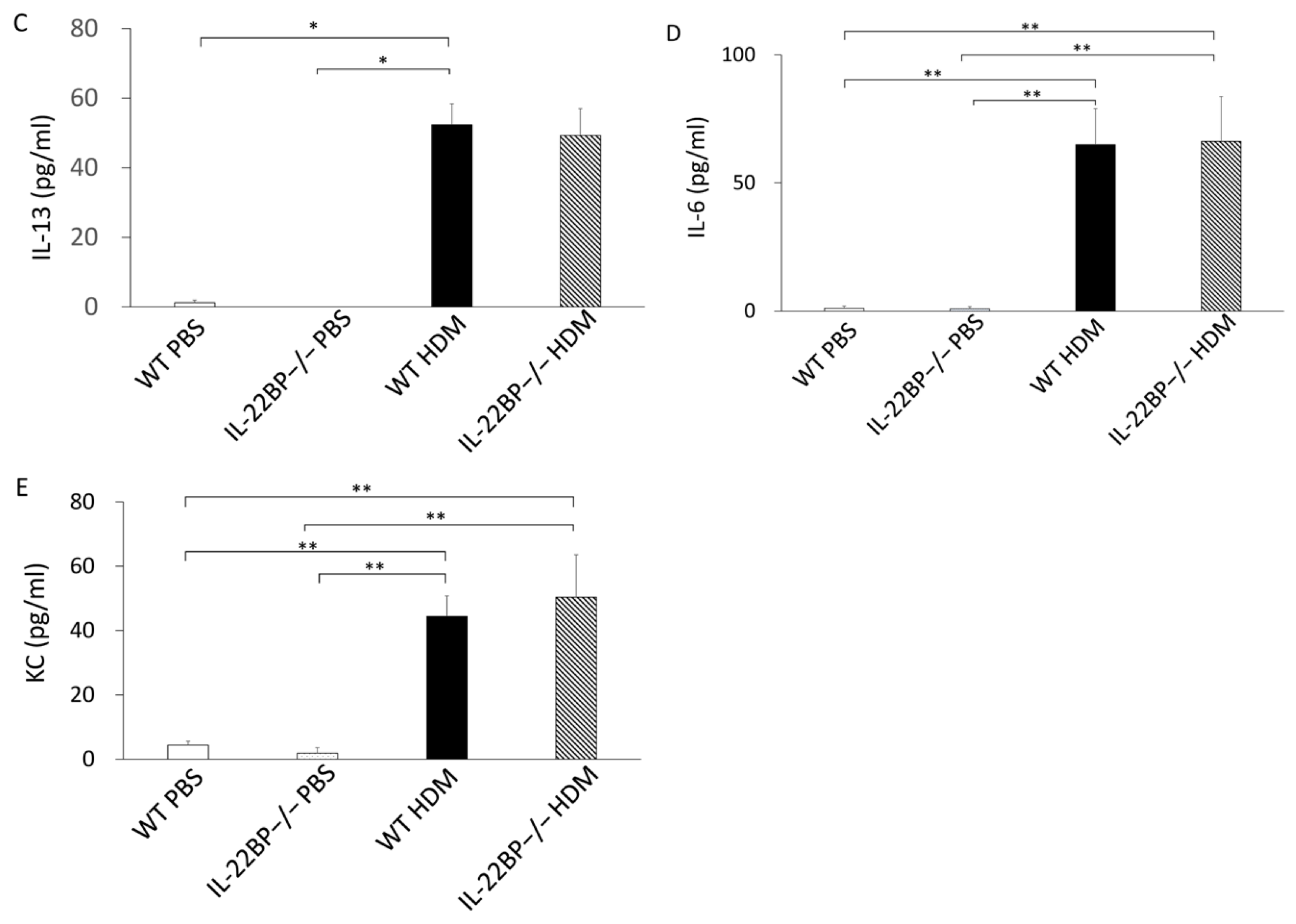
Cytokine and chemokine levels in BAL fluid of WT and IL-22BP−/− mice following sensitization and challenge. BAL fluid levels of (**A**) IL-4, (**B**) IL-5, (**C**) IL-13, (**D**) IL-6, and (**E**) KC were measured by ELISA, as described in Materials and Methods. Data are presented as means ± SE (*n* = 4–8 per group). * *p* < 0.05, ** *p* < 0.01. IL-22BP, interleukin-22 binding protein; WT, wild-type; SE, standard error; BAL, bronchoalveolar lavage; ELISA, enzyme-linked immuno-sorbent assay.

Sensitization and challenge with HDM extract significantly increased type 2 cytokines (IL-4, IL-5, and IL-13) in BAL fluid of WT mice; however, these cytokine levels did not differ between IL-22BP−/− mice and WT mice (Figure 4A–C). Similarly, inflammatory mediators such as IL-6 and KC were elevated after sensitization and challenge but showed no differences between the two groups (Figure 4D,E).

**Figure 5:**
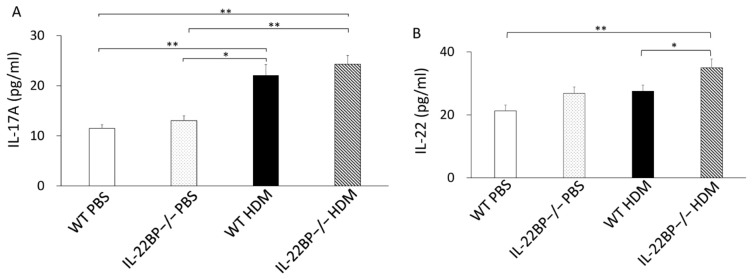
IL-17A and IL-22 levels in lung homogenates of WT and IL-22BP−/− mice following sensitization and challenge. (**A**) IL-17A and (**B**) IL-22 levels in lung homogenates were measured by ELISA, as described in Materials and Methods. Data are presented as means ± SE (*n* = 4–8 per group). * *p* < 0.05, ** *p* < 0.01. IL-22BP, interleukin-22 binding protein; WT, wild-type; SE, standard error; ELISA, enzyme-linked immunosorbent assay.

IL-17A levels in lung homogenates were increased in WT and IL-22BP−/− mice, with no significant intergroup differences (Figure 5A). Notably, IL-22 levels in lung homogenates of WT mice did not increase following sensitization and challenge, whereas IL-22 levels in IL-22BP−/− mice rose significantly and were significantly higher than those in WT mice under the same conditions (Figure 5B).

### 2.6. Cytokine Secretion from Splenic MNCs

**Figure 6:**
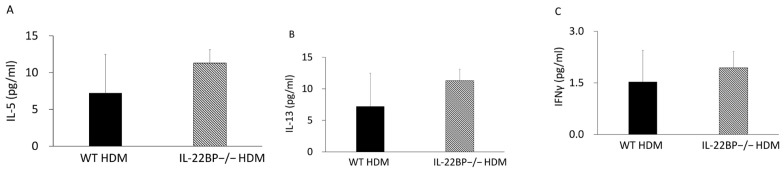
Cytokine production in splenic mononuclear cell cultures from WT/HDM and IL-22BP−/−/HDM mice. (**A**) IL-5, (**B**) IL-13, and (**C**) IFN-γ levels were measured as described in Materials and Methods. Data are presented as means ± SE. IL-22BP, interleukin-22 binding protein; WT, wild-type; SE, standard error; IFN, interferon; HDM, house dust mite.

To determine whether the attenuated AHR observed in IL-22BP−/− mice was attributable to impaired systemic type 2 cytokine production, we evaluated cytokine secretion from splenic MNCs in vitro. Upon HDM re-stimulation, IL-5, IL-13, and IFN-γ levels in IL-22BP−/− mice were not significantly different from those in WT mice (Figure 6). These findings suggest that IL-22BP deficiency does not impair systemic Th2 sensitization.

### 2.7. RhoA Protein Expression

**Figure 7:**
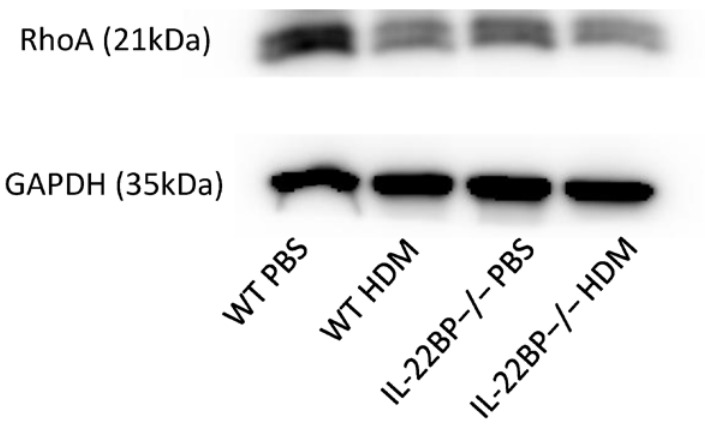
RhoA protein expression in lung homogenates of WT and IL-22BP−/− mice. RhoA protein levels were analyzed by Western blotting in WT/PBS, IL-22BP−/−/PBS, WT/HDM, and IL-22BP−/−/HDM groups. GAPDH was used as a loading control (lower panels). Upper panels show representative blots: WN, WT/PBS; KN, IL-22BP−/−/PBS; WP, WT/HDM; KP, IL-22BP−/−/HDM. IL-22BP, interleukin-22 binding protein; WT, wild-type; HDM, house dust mite; GAPDH, glyceraldehyde-3-phosphate dehydrogenase.

Previous studies have demonstrated that RhoA protein expression in the airways is increased during the development of airway hyperresponsiveness (AHR) [13]. To investigate whether IL-22BP affects RhoA protein expression, we evaluated its expression following HDM sensitization and challenge. However, no significant difference in RhoA protein expression was observed between the two groups after HDM sensitization and challenge (Figure 7). These findings suggest that, although IL-22BP contributes to the development of AHR, its effects are not mediated through the regulation of RhoA protein expression.

## 3. Discussion

IL-22BP functions as a soluble decoy receptor that neutralizes IL-22 activity. Previous studies have demonstrated that IL-22BP suppresses IL-22 signaling, which has been implicated in the pathogenesis of bronchial asthma [9]. These findings suggest a potential role of IL-22BP in allergic airway responses. However, its specific contribution to the development of such responses has not been systematically investigated and remains unclear.

We demonstrated for the first time that IL-22BP contributes to the development of allergen-induced AHR, a key feature of bronchial asthma. IL-22BP−/− mice exhibited significantly reduced AHR compared to WT mice following sensitization and challenge with HDM, and this reduction was associated with elevated IL-22 levels in the airway. These findings suggest that IL-22BP plays a pathogenic role in allergen-induced AHR and emphasize its potential as a novel therapeutic target for allergen-induced AHR.

T cells, particularly Th2 cells that release IL-4, IL-5, and IL-13, play pivotal roles in the development of AHR and eosinophilic inflammation [1,2,3]. By contrast, the contribution of Th17 cells, which secrete IL-17 and IL-22, to allergic airway responses remains unclear. In our study, using a murine model of HDM-induced allergy, we demonstrated that IL-22BP deficiency attenuated AHR compared to WT mice, and this effect was associated with increased IL-22 production in the airway. These findings suggest that elevated IL-22 suppresses the development of HDM-induced AHR in the absence of IL-22BP, which normally downregulates IL-22 activity. Consistent with our findings, several studies have reported a protective role of IL-22 against AHR [7,14,15]. Takahashi et al. [14] showed that inhibition of IL-22 significantly enhanced AHR following sensitization and challenge with OVA, and Taube et al. [15] reported that IL-22BP−/− mice exhibited greater AHR compared with WT mice after OVA sensitization and challenge. In those studies, intraperitoneal sensitization and airway challenge with OVA antigen were used. By contrast, other murine models of asthma have reported pro-inflammatory effects of IL-22, including enhanced AHR, in which subcutaneous sensitization was used [8,9,16]. Therefore, differences in the route of sensitization may account for these contrasting outcomes, with intraperitoneal sensitization favoring a protective effect of IL-22 and subcutaneous sensitization favoring pro-inflammatory effects. Notably, Ito et al. [7] reported that IL-22BP−/− mice exhibited higher AHR compared to WT mice when sensitized and challenged with HDM, a clinically relevant protease allergen, supporting a protective role of IL-22 consistent with our findings. In their study, sensitization and challenge were performed via the airway, consistent with our findings. Taken together, these findings suggest that airway sensitization and challenge with the protease allergen HDM, which closely reflects human asthma, may result in IL-22-mediated suppression of AHR. Therefore, enhancing IL-22 activity through inhibition of IL-22BP may represent a useful strategy to attenuate allergen-induced AHR.

In our study, IL-22BP−/− mice, which completely lack IL-22BP, exhibited increased airway IL-22 levels and reduced AHR compared to WT mice following sensitization and challenge, indicating a suppressive role of IL-22 in AHR development. By contrast, WT mice exhibited elevated IL-22BP levels in the airway, which were associated with enhanced AHR but did not correspond to decreased IL-22 levels. The mechanism underlying this discrepancy remains unclear. It is possible that increased IL-22BP does not directly alter IL-22 concentrations, whereas a reduction or complete absence of IL-22BP may lead to upregulation of IL-22 in the airway.

In our study, airway inflammation, including eosinophilic infiltration and goblet cell metaplasia, did not differ significantly between IL-22BP−/− and WT mice following HDM sensitization and challenge. Previous studies have suggested that the suppressive role of IL-22 on AHR is mediated through the inhibition of airway inflammation and type 2 cytokine production [7]. The reason for the lack of differences in airway inflammation and type 2 cytokine levels between IL-22BP−/− and WT mice remains unclear. It is possible that enhanced IL-22 activity resulting from IL-22BP deficiency preferentially downregulates AHR without substantially affecting airway inflammation. Further studies are needed to clarify this discrepancy.

Upregulation of RhoA protein in bronchial smooth muscle has been reported in allergic airway inflammation, where RhoA-mediated Ca^2+^ sensitization enhances smooth muscle contraction and contributes to the development of airway hyperresponsiveness (AHR) [13]. To further investigate the mechanisms underlying the attenuated AHR observed in IL-22BP-deficient mice, we assessed RhoA protein expression following HDM sensitization and challenge. However, RhoA protein expression was comparable between IL-22BP-deficient and WT mice. These findings suggest that, although IL-22BP contributes to the development of AHR, its effects are unlikely to be mediated through the regulation of RhoA protein expression. The finding that IL-22BP deficiency attenuated AHR without affecting RhoA protein expression suggests that the IL-22/IL-22BP axis may regulate airway responses through mechanisms distinct from those associated with altered RhoA expression. Given the established roles of IL-22 in epithelial protection, tissue repair, and maintenance of mucosal homeostasis, modulation of IL-22BP may represent a broader therapeutic approach for airway diseases beyond the regulation of AHR.

The critical role of IL-22BP in acute lung injury has recently been reported [12]. In that study, IL-22BP−/− mice exhibited greater weight loss, increased mortality, and enhanced pulmonary inflammation during the acute phase compared to WT controls, and the inflammation was driven by excess IL-22 production. These findings suggest that IL-22BP suppresses the development of acute lung injury. Therefore, while inhibition of IL-22BP may attenuate AHR, it could potentially exacerbate acute lung injury. Further studies are needed to clarify the role of IL-22BP, particularly in the context of asthma associated with acute lung injury.

IL-22BP is highly expressed in secondary lymphoid organs, including the spleen and lymph nodes [10]. It is predominantly secreted by dendritic cells and is constitutively expressed by conventional DCs under steady-state conditions; however, its expression is downregulated during inflammation, enhancing IL-22 bioactivity [16]. Recent studies have demonstrated that IL-22BP can be produced by neural, immune, and structural cells [17]. Moreover, CD4^+^ T cells and eosinophils have been shown to play a pathogenic role in inflammatory bowel disease by antagonizing the protective effects of IL-22 through IL-22BP expression [18,19]. These findings raise the possibility that IL-22BP produced by DCs, CD4^+^ T cells, or eosinophils may act as an endogenous inhibitor of IL-22 in the development of AHR.

In conclusion, we demonstrated that IL-22BP expression in the airways of WT mice increased following sensitization and challenge with HDM allergen. IL-22BP−/− mice exhibited lower AHR compared to WT mice, which was associated with elevated IL-22 levels in the airway. These findings suggest that targeting IL-22BP may represent a potential therapeutic strategy for controlling allergen-induced AHR.

## 4. Materials and Methods

### 4.1. Animals

IL-22BP-deficient mice (B6-Il22ra2tm1.1Ohno) were generated as reported previously [20]. The mutant strain was originally established from E14.1 embryonic stem cells on a 129 genetic background. After generation of bone marrow chimeras, mice were crossed with CD-1 Cre-transgenic animals expressing Cre recombinase transiently in oocytes. The resulting offspring were backcrossed onto the C57BL/6J background for nine generations. Female knockout mice and age-matched C57BL/6J wild-type mice (8–12 weeks old) were used throughout the study. The mutant mice displayed normal viability and fertility and did not develop any apparent spontaneous abnormalities under specific pathogen-free housing conditions. All procedures were conducted in accordance with NIH guidelines and were approved by the Animal Care and Use Committee of Okayama University.

### 4.2. Experimental Protocol (Airway Sensitization and Challenge)

Allergic airway inflammation was induced using a house dust mite (HDM) model as previously reported [21]. Mice received intranasal sensitization with 25 μg HDM extract dissolved in 30 μL PBS once daily on days 0, 1, and 2. Airway challenge was subsequently performed with 5 μg HDM extract in 30 μL PBS on days 14–17. Twenty-four hours after the last challenge, airway responsiveness was measured and biological samples were collected. For dose–response experiments, wild-type mice were sensitized with either 15 μg or 25 μg HDM before challenge with 5 μg HDM.

### 4.3. Determination of Airway Responsiveness

Airway responsiveness was quantified using a FlexiVent™ system (SCIREQ, Montreal, Canada) [21]. Mice were anesthetized with ketamine (80 mg/kg) and xylazine (10 mg/kg) administered intraperitoneally, followed by tracheostomy and connection to a mechanical ventilator. Lung resistance was then measured after exposure to increasing concentrations of aerosolized methacholine. Baseline respiratory parameters were comparable among all experimental groups.

### 4.4. Bronchoalveolar Lavage (BAL)

Following measurement of airway responsiveness, bronchoalveolar lavage (BAL) was performed through the tracheal cannula using two sequential instillations of 1 mL prewarmed Hanks’ balanced salt solution. Recovered lavage fluid was collected and total leukocyte numbers were determined. Differential cell counts were obtained from May–Giemsa-stained cytospin preparations by examining at least 200 cells per specimen in a blinded fashion [22].

### 4.5. Lung Histology

Lung specimens were fixed with 10% neutral-buffered formalin and embedded in paraffin after sectioning around the main bronchial region. Histological sections were stained with hematoxylin and eosin or periodic acid–Schiff reagent. Goblet cell hyperplasia was quantified in more than ten bronchioles per mouse. Measurements of epithelial length along the basement membrane and airway luminal area were performed using ImageJ software (version 1.54, National Institutes of Health, Bethesda, MD, USA) [23].

### 4.6. Lung Homogenates

After sacrifice, lung tissues were rapidly harvested and stored at −80 °C until analysis. Samples were homogenized in PBS supplemented with 0.1% Triton X-100 and a protease inhibitor cocktail. Following centrifugation at 14,000 rpm for 30 min, supernatants were collected and used for subsequent cytokine measurements [24].

### 4.7. Culture of Splenic Mononuclear Cells (MNCs)

Splenic mononuclear cells (MNCs) were isolated from sensitized and challenged mice 24 h after the final HDM exposure. Spleens were mechanically dissociated and cells were separated by Histopaque density-gradient centrifugation. After washing, cells were suspended in RPMI-1640 medium containing 10% heat-inactivated fetal calf serum and antibiotics. Cells (4 × 10^5^/well) were cultured in 96-well plates with or without HDM extract (10 μg/mL). Culture supernatants were harvested after 48 h and analyzed for cytokine production [21].

### 4.8. Measurement of Cytokines and Chemokines

Concentrations of cytokines and chemokines were determined using commercially available ELISA kits according to the manufacturers’ protocols. BAL fluid was analyzed for IL-4, IL-5, IL-6, IL-13, and KC, whereas IL-17A and IL-22 were measured in lung tissue homogenates. IL-22BP concentrations were assessed in both BAL fluid and serum samples. Most ELISA reagents were purchased from R&D Systems, while the IL-22BP assay kit was obtained from MyBioSource [24].

### 4.9. Immunohistochemistry for IL-22BP

Paraffin-embedded lung sections were processed using a Bond Max automated staining platform (Leica Biosystems). Sections were incubated with a mouse monoclonal antibody against IL-22BP (ab203211, Abcam; 1:200 dilution). Positive staining was evaluated microscopically at high magnification (×400).

### 4.10. Assessment of RhoA Expression

RhoA protein expression was examined by Western blot analysis as described previously [13]. Equal amounts of protein were separated by SDS-PAGE and transferred onto membranes. After blocking, membranes were incubated with a rabbit monoclonal anti-RhoA antibody (Cell Signaling Technology). Protein bands were visualized using enhanced chemiluminescence and quantified densitometrically. GAPDH was used as a loading control to ensure equal protein loading across samples. Western blot experiments were independently repeated at least three times to confirm reproducibility.

### 4.11. Statistical Analysis

Results are expressed as mean ± SEM. Analysis of variance was used to determine differences among groups. When significant differences were detected, post hoc multiple-comparison analyses were performed using the Tukey–Kramer test. Student’s *t*-test was used for parametric comparisons between two groups, whereas the Mann–Whitney U test was used for nonparametric data. A *p* value < 0.05 was considered statistically significant.

## Data Availability

The data presented in this study are available on request from the corresponding author.

## Supplementary Materials

The following supporting information can be downloaded at: https://www.mdpi.com/article/10.3390/ijms27135909/s1.

## Abbreviations

The following abbreviations are used in this manuscript:

AHR	Airway hyperresponsiveness
APC	Antigen-presenting cell
BAL	Bronchoalveolar lavage
DC	Dendritic cell
HE	Hematoxylin and eosin
HDM	House dust mite
IFN	Interferon
IL	Interleukin
ILC	Innate lymphoid cell
Mch	Methacholine
MNC	Mononuclear cell
NIH	National Institutes of Health
OVA	Ovalbumin
PAS	Periodic acid–Schiff
RL	Lung resistance
Th1	T helper type 1
Th2	T helper type 2
TSLP	Thymic stromal lymphopoietin
