# Supplementary figures and images for: Contribution of Interleukin-22 Binding Protein to the Development of Allergen-Induced Airway Hyperresponsiveness
Source: Int J Mol Sci. 2026 Jun 30;27(13):5909. doi: 10.3390/ijms27135909 (PMC13361637; doi:10.3390/ijms27135909)

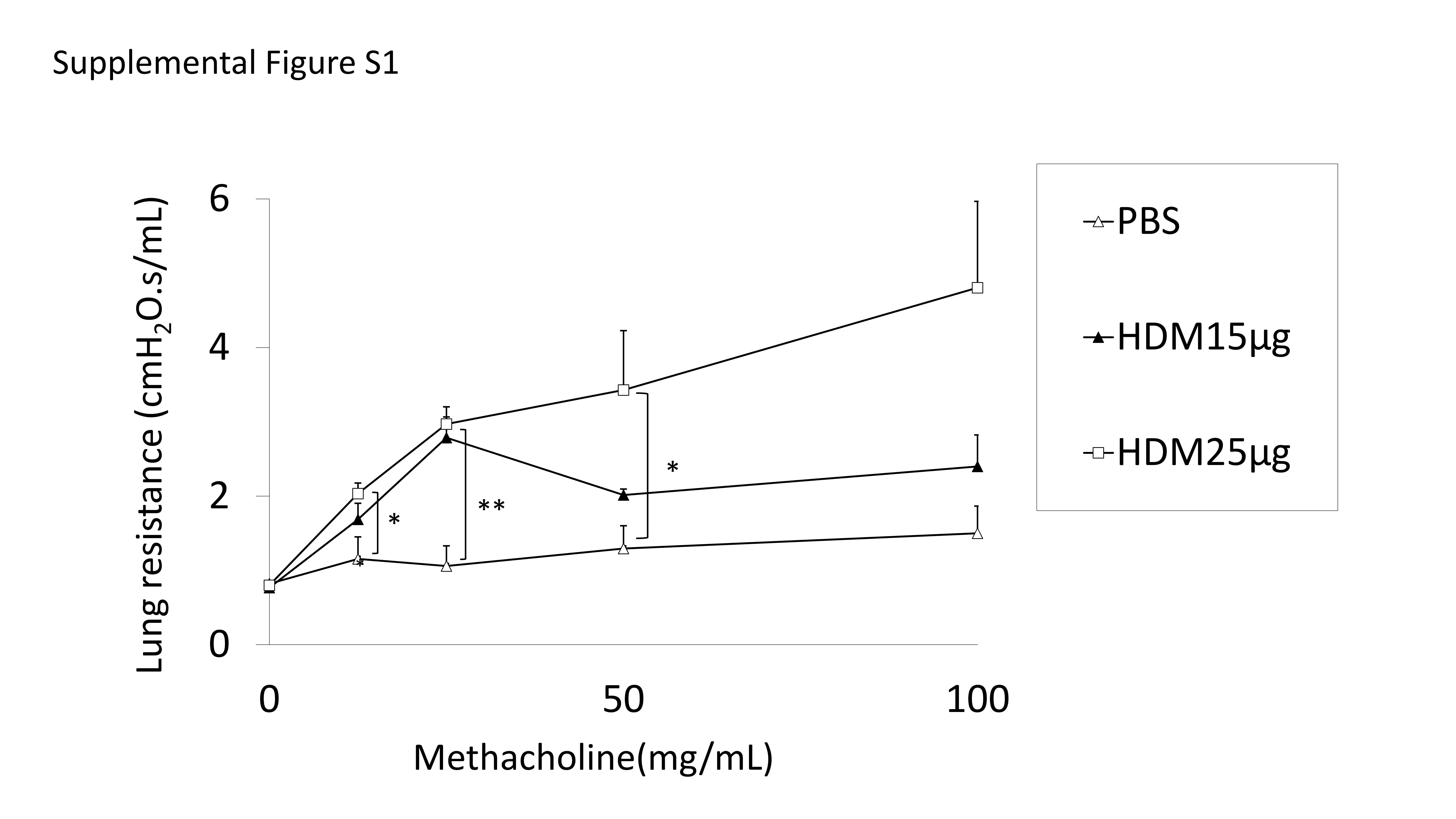

Supplement: Supplementary file 1 [file ijms-27-05909-s001.zip › Supplemental_Figure_S1_300dpi(2).tif]

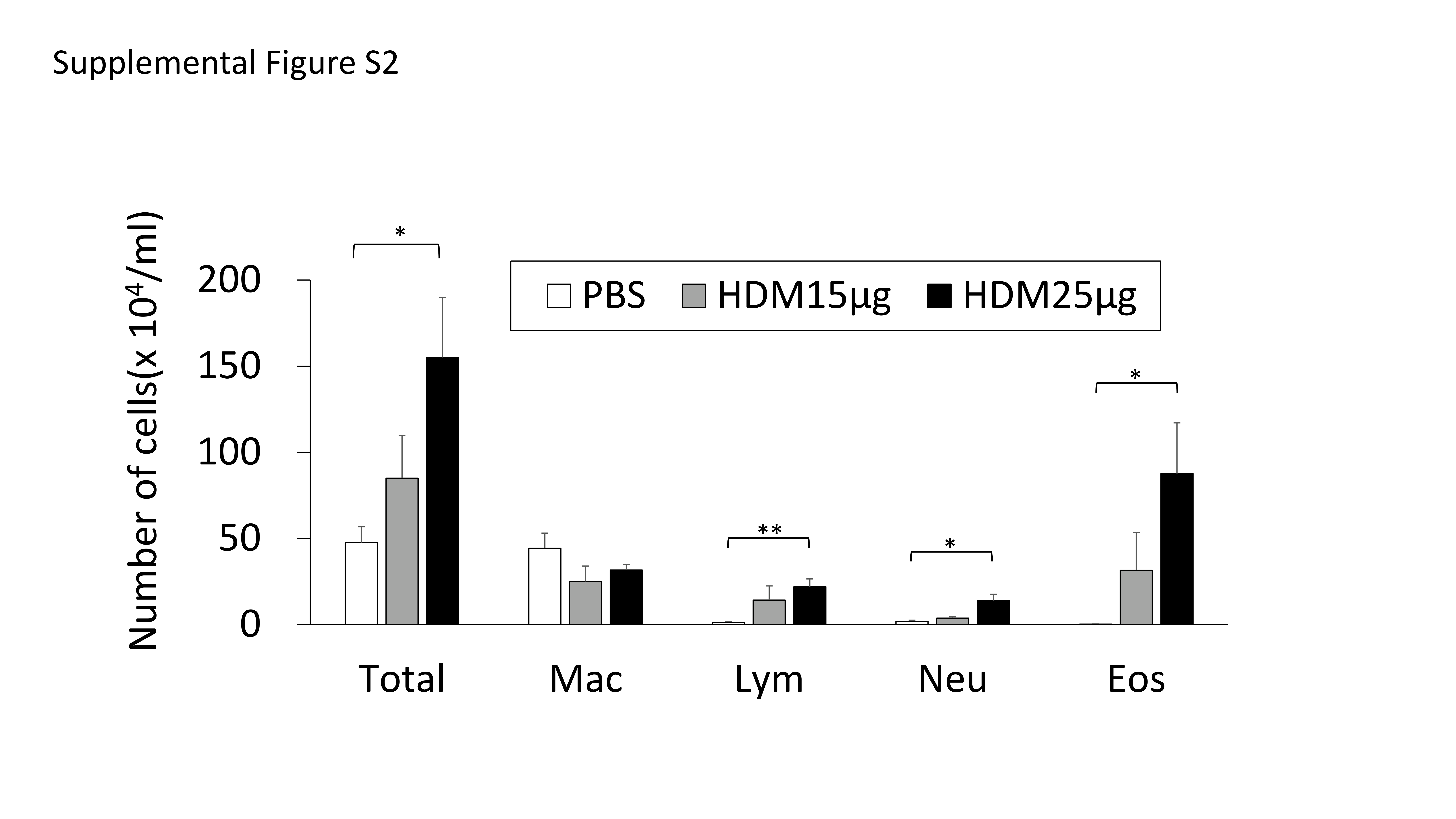

Supplement: Supplementary file 1 [file ijms-27-05909-s001.zip › Supplemental_Figure_S2_300dpi(2).tif]

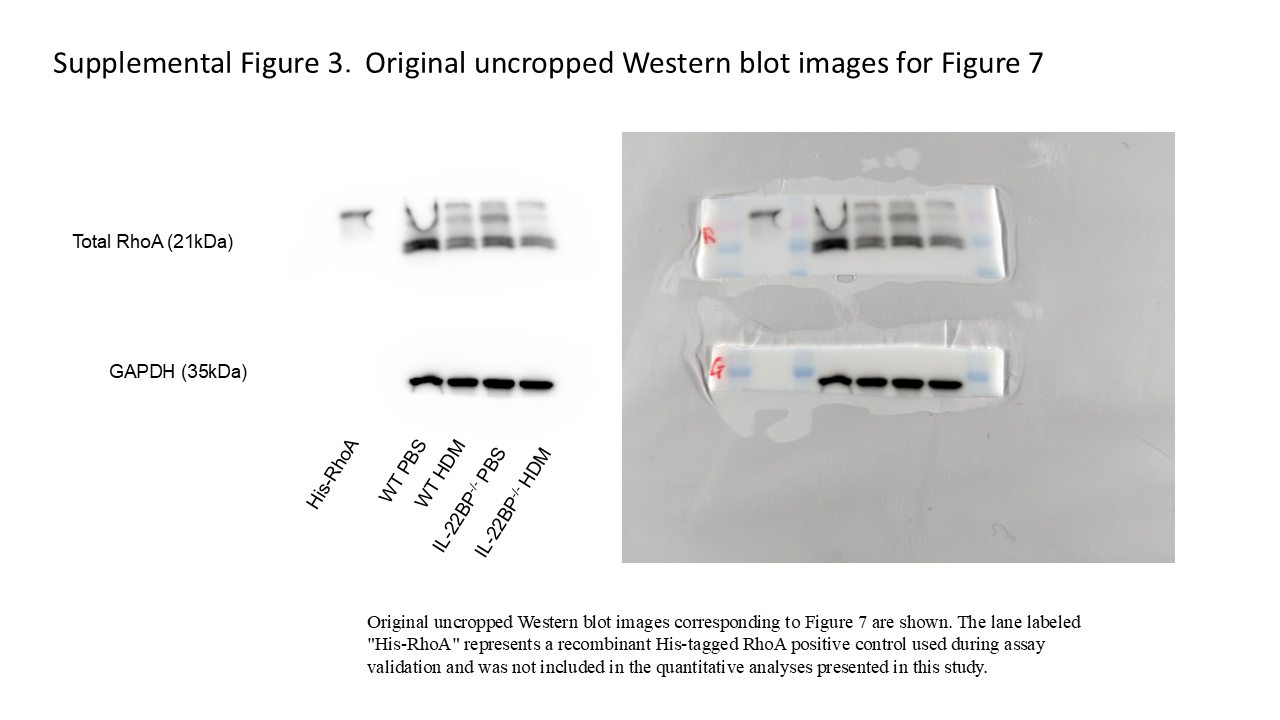

Supplement: Supplementary file 1 [file ijms-27-05909-s001.zip › Supplemental_Figure_S3.TIF]
